# Repurposing Niclosamide as a plausible neurotherapeutic in autism spectrum disorders, targeting mitochondrial dysfunction: a strong hypothesis

**DOI:** 10.1007/s11011-023-01247-x

**Published:** 2023-06-07

**Authors:** Manasi Varma, Ranjana Bhandari, Anurag Kuhad

**Affiliations:** Pharmacology Research Laboratory, UGC- Centre of Advanced Study, University Institute of Pharmaceutical Sciences, Panjab University, Chandigarh, 160 014 India

**Keywords:** Autism spectrum disorders, Niclosamide, ERK/MAPK, Mitochondrial dysfunction, Neuroinflammation

## Abstract

Autism Spectrum Disorders (ASD) are a complex set of neurodevelopmental manifestations which present in the form of social and communication deficits. Affecting a growing proportion of children worldwide, the exact pathogenesis of this disorder is not very well understood, and multiple signaling pathways have been implicated. Among them, the ERK/MAPK pathway is critical in a number of cellular processes, and the normal functioning of neuronal cells also depends on this cascade. As such, recent studies have increasingly focused on the impact this pathway has on the development of autistic symptoms. Improper ERK signaling is suspected to be involved in neurotoxicity, and the same might be implicated in autism spectrum disorders (ASD), through a variety of effects including mitochondrial dysfunction and oxidative stress. Niclosamide, an antihelminthic and anti-inflammatory agent, has shown potential in inhibiting this pathway, and countering the effects shown by its overactivity in inflammation. While it has previously been evaluated in other neurological disorders like Alzheimer’s Disease and Parkinson’s Disease, as well as various cancers by targeting ERK/MAPK, it’s efficacy in autism has not yet been evaluated. In this article, we attempt to discuss the potential role of the ERK/MAPK pathway in the pathogenesis of ASD, specifically through mitochondrial damage, before moving to the therapeutic potential of niclosamide in the disorder, mediated by the inhibition of this pathway and its detrimental effects of neuronal development.

## Introduction

**Fig. 1:**
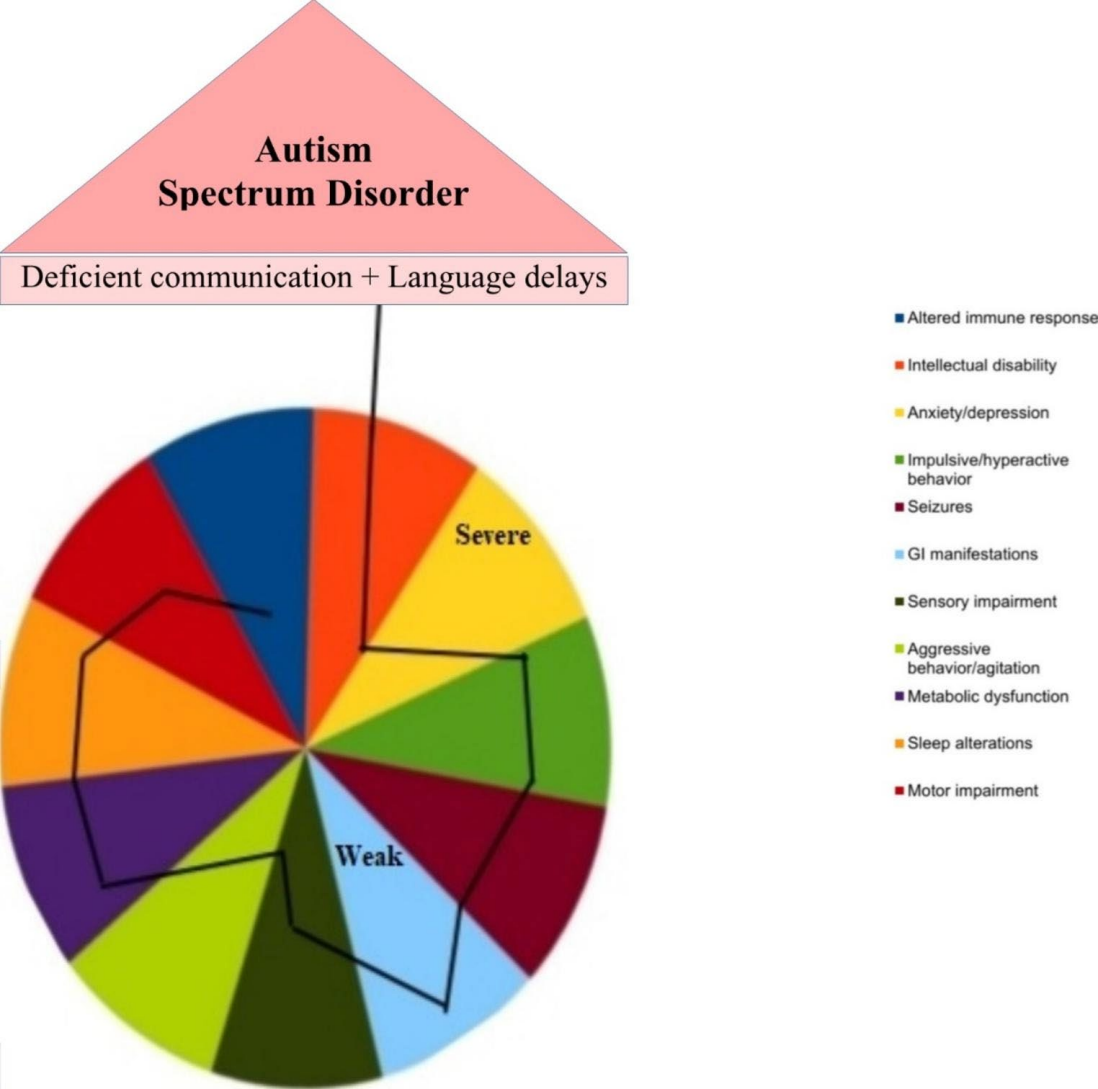
Hallmark symptoms of ASD. Autism encompasses a number of interlinked symptoms, and the degree of their severity varies depending on which end of the spectrum the patient lies at. The two hallmarks of the disorder, which are often used to characterize its presence, are language delays and defects in communication

Autism spectrum disorder, often contracted to ASD, is a heterogeneous set of neurodevelopmental deficits, primarily affecting children and characterized according to the Diagnostic and Statistical Manual of Mental Disorders-V (DSM-V), on the basis of either lacking “social communication,” or “restricted, repetitive and/or sensory behaviors or interests” (Fig. 1). As per the data published by World Health Organization (WHO) in 2022, 1 in 100 children are affected by ASD worldwide (World Health Organization, 2022). The etiology of ASD is not very well understood, but a complex interplay of genetic and environmental factors (including prenatal) has been implicated through several studies done on this front. Till date, as many as 100 genes and more than 50 copy number variants (CNVs, different number of tandem repeats of genome sections) have been linked to the risk of developing ASD (Betancur 2011). Additional studies have further shed light on possible association of autism with over 1000 genes and 2000 CNVs, but the strength is variable (Simmons Foundation, 2018). Since single gene analysis of such large numbers of genes is faced with multiple complexities, there has been a shift towards focusing on specific signaling pathways that are believed to play a role in the pathogenesis of autism, and where these genes are known to converge (Levitt and Campbell 2009). Among these, the extracellular signal-regulated kinase/mitogen activated protein kinase (ERK/MAPK) pathway has received significant attention towards a possible link to ASD.

The ERK/MAPK pathway is known to interact with multiple genes that have been implicated in autism, and genome-wide association analysis of the same have supported these findings. As such, a dysregulation of this pathway has been found to result in many CNS disorders, including ASD-related syndromes, in many studies. These syndromes are collectively known as Rasopathies, due to the fact that the affected genes include those encoding for elements which function together with Ras, a G-protein responsible for activating ERKs (Levitt and Campbell 2009; Tidyman and Rauen 2009). It has been found that ASD is linked to the occurrence of many Rasopathies, and there have been multiple reports suggesting the possible relation of ERK/MAPK pathway defects with the incidence of ASD (Vithayathil et al. 2018; Aluko et al. 2021)⁠⁠. Moreover, a detailed study has found that single nucleotide polymorphisms (SNPs) in the ERK/MAPK-related genes are more common in subjects presenting with idiopathic ASD (Mitra et al. 2017; Nisar et al. 2019).

Niclosamide is an FDA-approved antihelminthic drug which is routinely used to treat tapeworm infections by inhibiting their mitochondrial oxidative phosphorylation and ATP production. In addition, it has long been known to have significant immunomodulating activity, and has been shown to inhibit a number of signaling pathways, including the Wingless-related integration site (Wnt)/β-catenin, nuclear factor kappa B (Nf-κB), signal transducer and activator of transcription 3 (STAT3), and mammalian target of rapamycin (mTOR) (Chen et al. 2018). However, while these targets are known to be rather well-characterized in terms of the effect that niclosamide has on them, there are also other targets, including the phosphoinositode 3 kinase/Akt (PI3K/Akt) and ERK/MAPK pathways, that are seen to be downregulated by the agent. Hence, given the possible relation of the ERK pathway in autism, there has been interest in the potential role of niclosamide in the management of the prognosis of ASD. This article aims to discuss the possible therapeutic benefit of niclosamide in the treatment of autism spectrum disorders.

## The MAPK/ERK pathway: basics

Mitogen activated protein kinases (MAPKs) are a family of protein kinases that are activated upon dual phosphorylation of their tyrosine and threonine residues (Roux and Blenis 2004). The ERK subfamily are among the most well-established examples, along with others such as the c-JUN N-terminal kinases (JNKs) and the p38 kinases.

The ERK/MAPK pathway was the first to be elucidated, in the form of the microtubule associated protein 2 (MAP2), which was found to be involved in the functioning of insulin by Thomas Sturgill and Brian Ray, some 34 years ago (Ray & Sturgill, 1988).

**Fig. 2:**
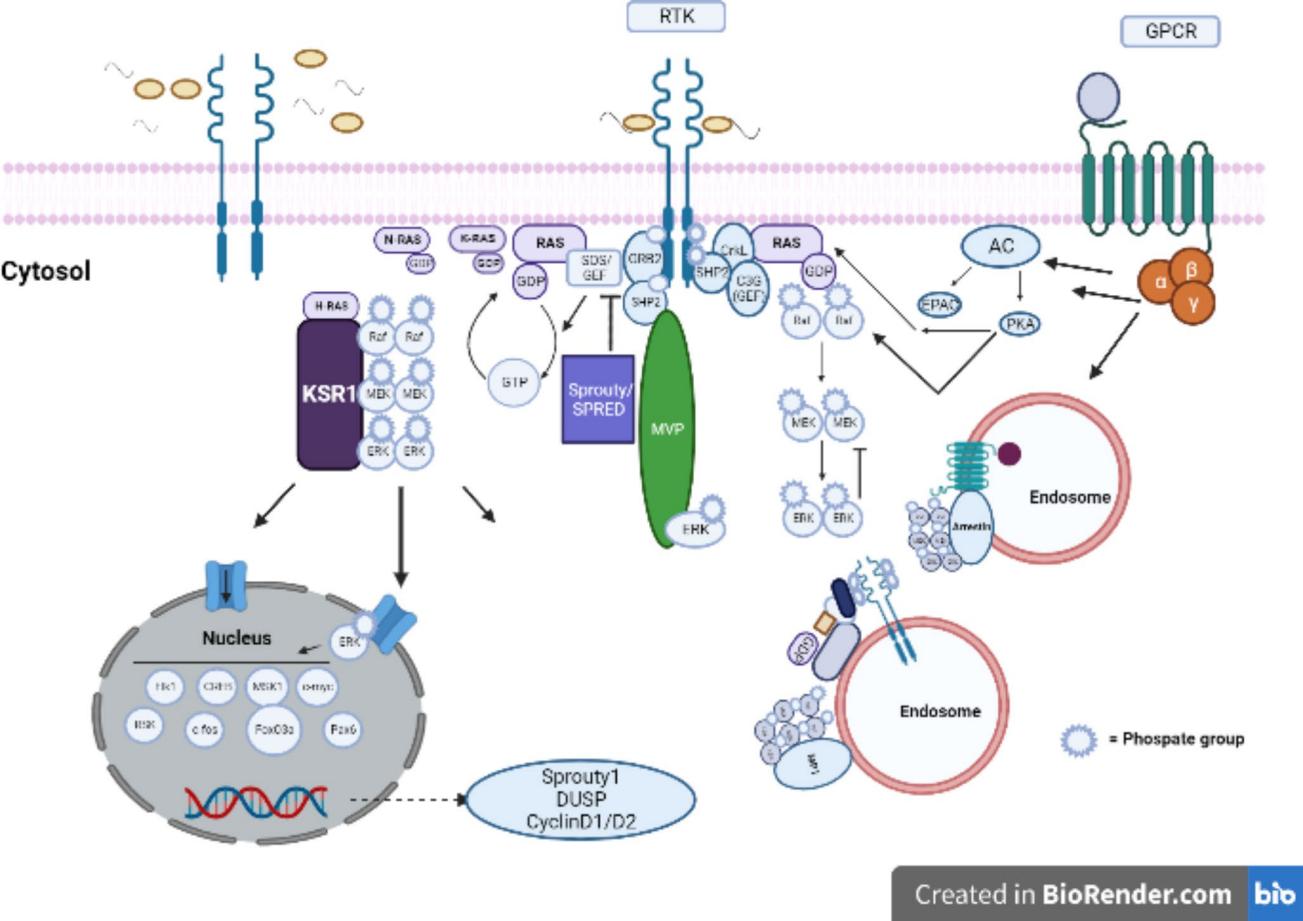
Components of the ERK/MAPK pathway. The activation of MAPK pathway begins when an external ligand binds to the tyrosine kinase receptors, such as fibroblast growth factor (FGF) with fibroblast growth factor receptor (FGFR). This causes dimerization and activation of the receptor tyrosine kinases (RTK), which then results in phosphorylation of molecules like growth factor receptor-bound protein (Grb2) and Src homology region 2 containing protein tyrosine phosphatase 2 (SHP2). The latter is linked to rat sarcoma virus (Ras) through son of sevenless (SOS)/guanine nucleotide exchange factors (GEF), which brings about the phosphorylation of rapidly accelerated fibrosarcoma (Raf), mitogen activated protein kinase kinase (MEK) and finally, ERK. These downstream mediators can enter the nucleus and induce the translation of multiple genes. Ras-proxitmate-1 (Rap1) also associated with the RTK, and through a similar phosphorylation of downstream molecules, can interact with endosomes. Finally, this Rap1 can also be activated by G-protein coupled receptors (GPCRs), resulting in a similar effect

Subsequently dubbed “mitogen-activated protein kinase,” MAPK was purified and resolved into two isoforms, known as ERK1 and ERK2 (Cobb and Goldsmith 1995). They essentially link growth factor-mediated signals to intracellular pathways via the activation of receptor tyrosine kinases (RTKs) or occasionally G-protein coupled receptors (GPCRs) (Schlessinger 1988, 2000). The final step of this activation is brought about by Ras, often in association with another molecule known as Raf (Pawson 2004; Hibbing et al., 2011).

The ERK/MAPK cascade is regulated by a complex set of factors (Fig. 2), including both temporal and spatial regulation. This is mainly orchestrated by Ras, as well as a different regulatory molecule known as Rap1 (Baass et al. 1995; Gureasko et al. 2008), often through the activity of certain scaffolding proteins (Kornfeld et al., 1995) and nuclear export sequences (Formstecher et al. 2001). The temporal regulation is of particular importance, especially in terms of neuronal health, since these signaling molecules have been found to largely localize during three phases of neuronal development, namely the embryonic development phase, the early postnatal phase, and the adulthood and maturation phase. Among these, the first two phases are of special significance when considering neurodevelopmental disorders such as autism spectrum disorders, while the third phase is often implicated in degenerative disorders like Alzheimer’s and Parkinson’s Disorders (Kim and Choi 2010).

### ERK/MAPK signaling in CNS disorders

While the proper functioning of the ERK system has been implicated in the development of synaptic networks and the normal growth of the brain, any impairment in the signaling by this cascade can lead to changes that proceed in one of two ways, both ultimately giving rise to a negative outcome. It has been found that while under-expression and reduced performance of the ERK pathway can bring about inadequate plasticity of the synapses, over-expression and elevated activity has been associated with a different set of problems altogether.

The role of ERK in neurodegeneration is worth mentioning here, because there exists some evidence for the line of thought ASD might be linked to at least a certain level of degeneration in different regions of the brain (Kern et al. 2013). This evidence mainly includes 1) loss of neuronal cells, 2) presence of pro-inflammatory cytokines, 3) activation of microglia and astrocytes, 4) presence of oxidative stress, and 5) increased levels of 8-oxo-guanosine levels. Overactivity of this pathway has been linked to certain neurodegenerative disorders as well (Colucci-D’ Amato et al., 2003).

For example, considering the presence of tau in the brains of patients of Alzheimer’s Disease, ERK is involved in the phosphorylation of this protein (Kim and Choi 2010). Moreover, MAPK signaling is an active part of various mechanisms occurring in Alzheimer’s Disease, including neuronal apoptosis, oxidative stress, β- and γ-secretase activation at multiple levels, and stabilization of amyloid precursor protein (APP) via phosphorylation (Muresan and Muresan 2007).

At the same time, the story in Parkinson’s Disease (PD) is different in certain respects (Kim and Choi 2010). α-Synuclein, which is an important marker present in the brains of PD patients, is believed to activate the ERK pathway among others in microglial cells (Su et al. 2008), and this causes the release of tumor-necrosis factor alpha (TNF-α) and interleukin-1 beta (IL-1β), bringing about an inflammation in the region, which might in turn be responsible for the neurodegeneration observed.

Another line of evidence could potentially hint towards the origin of the repetitive actions that are another hallmark trait of most types of autisms. According to the results of a study reported in 2017 (Ullrich et al. 2018), upregulated expression and functioning of the brain derived neurotrophic factor (BDNF)/receptor tyrosine kinase B (TrkB)/ERK-MAPK system (Guiton et al. 1994; Huang and Reichardt 2001, 2003)⁠ especially in the circuits of the thalamo-amygdala region, might be responsible for the observation of obsessive-compulsive disorder-like behaviors (OCD). Since signs of inflammation are observed in most of these manifestations, there is reason to believe that this pathway has a potential role in neuroinflammation, which is marked by the presence of oxidative stress often brought about by mitochondrial dysfunction.

Nevertheless, the actual importance of neurodegeneration owing to oxidative stress in the development of ASD and its related symptoms is the subject of major debate, with arguments being made both in favor of and against its role in the disorder.

## ERK signaling and mitochondrial dysfunction

While there are a number of ways in which improper functioning of the ERK pathway can lead to disruptions in downstream signaling, neuronal health, and synaptic plasticity, one impact that is of particular interest is on the mitochondria. There have been multiple studies that have suggested a potential link between mitochondrial dysfunction and over- or derailed- activity of the ERK/MAPK cascade.

The results of a few such studies (He and Aizenman 2010)⁠ have reported that ERK 1/2 functioning depends on a critical balance between the phosphorylation (activation) and dephosphorylation (inactivation) of this molecule, while also suggesting that prolonged or enhanced activation can lead to cell death in a zinc-dependent fashion by inducing oxidative stress-like conditions.

Zinc is a known neurotoxin, and increasing evidence has been put forth regarding its potential to bring about apoptotic or non-apoptotic cell death (Cheung and Slack 2004)⁠ in relation to ERK abnormalities. It is believed that increased levels of this metal can lead to a higher degree of ERK activation in a Ras-dependent manner. Other studies have found that this overactivation can in turn give rise to oxidative stress (Yagoda et al. 2007)⁠ mediated by the activation of voltage-gated anion channels in the mitochondrial membranes in non-neuronal cells.

More interestingly, one study has even found that while the ERK/MAPK system is a key mediator of the zinc-mediated mitochondrial dysfunction model, stimulating the production of reactive oxygen species (ROS) and causing oxidative stress, ROS too can cause activation of ERK 1/2 (Samavati et al. 2002).

Another line of evidence for this relationship takes a reverse approach, suggesting that inhibition of this signaling pathway can alleviate the symptoms of mitochondrial dysfunction in disorders such as Alzheimer’s Disease (Gan et al. 2014). Moreover, it has also been found that antioxidant treatment, which is a well-described therapy to oxidative stress induced by improper activity of the mitochondria, protects against mitochondria/ROS-mediated ERK activation.

So, it might be said that both ERK and ROS induce each other, and the activated ROS can lead to destructive changes inside the cell, while the activated ERK leads to other detrimental effects through different mechanisms.

Also, the results from a separate study have suggested that the apoptotic pathway mediated by mitochondria through the B-cell leukemia/lymphoma 2 (BCL-2) protein cascade is also under the direct influence of ERK, and a major hallmark of the activation of this pathway is the destruction of mitochondrial components. In this cascade, the BCL-2 family, an antagonistic set of pro-survival and pro-death proteins involved primarily in the apoptotic process, is crucial (Czabotar et al. 2014).

Initiation of apoptosis is brought about by pro-apoptotic signals which drive the release of cytochrome *c* into the cytosol through mitochondrial outer membrane permeabilization (MOMP). This then marks the beginning of the caspase apoptotic system (Tait and Green 2010), which eventually results in cell death. Interestingly, the regulation of both these types of BCL2 proteins has been found to be under the influence of ERK1/2.

As an example, the prosurvival proteins BCL2 and macrophage C-type lectin (MCL1) are under transcriptional regulation by cyclic adenosine monophosphate (cAMP)-responsive element binding protein (CREB), which in turn is activated following phosphorylation of ERK-dependent kinases, mitogen and stress-activated kinases (MSK) and ribosomal s6 kinase (RSK) (Townsend et al. 1999; Wilson et al. 1996). Moreover, the pro-apoptotic proteins (BCL-2 associated agonist of cell death (BAD), BCL-2 modifying factor (BMF), BCL-2 interacting mediator of cell death (BIM), BCL-2 interacting killer (BIK) and p53 upregulated modulator of apoptosis (PUMA) are under direct inhibition by ERK, while phorbol-12-myristate-13-acetate-induced protein 1 (also known as NOXA), a protein potentially involved in autophagy, is also expressed in response to the signaling molecule. This serves to show that the functioning of the mitochondria and also its possible dysfunction might be linked to the ERK pathway, further underlining the relation between this kinase and mitochondrial damage.

Dysfunctional mitochondria are routinely eliminated from the cell through a process known as mitophagy, which has been noted, through multiple studies, to be influenced by ERK signaling (Dagda et al. 2009). Mutations in MEK or ERK2 have, for example, an inductive effect on mitophagy, while a knockout of the latter, or inhibition of MEK by U0126, brings about a blockade of mitophagy in response to hypoxia and starvation (Hirota et al. 2015). And due to the link between mitochondrial fission and mitophagy, ERK has also been implied to have a role in the former, a hypothesis that has been put forward in diseases like Alzheimer’s Disease. This further consolidates the correlation between ERK processing and mitochondrial damage.

### Mitochondrial dysfunction and autism spectrum disorders

**Fig. 3:**
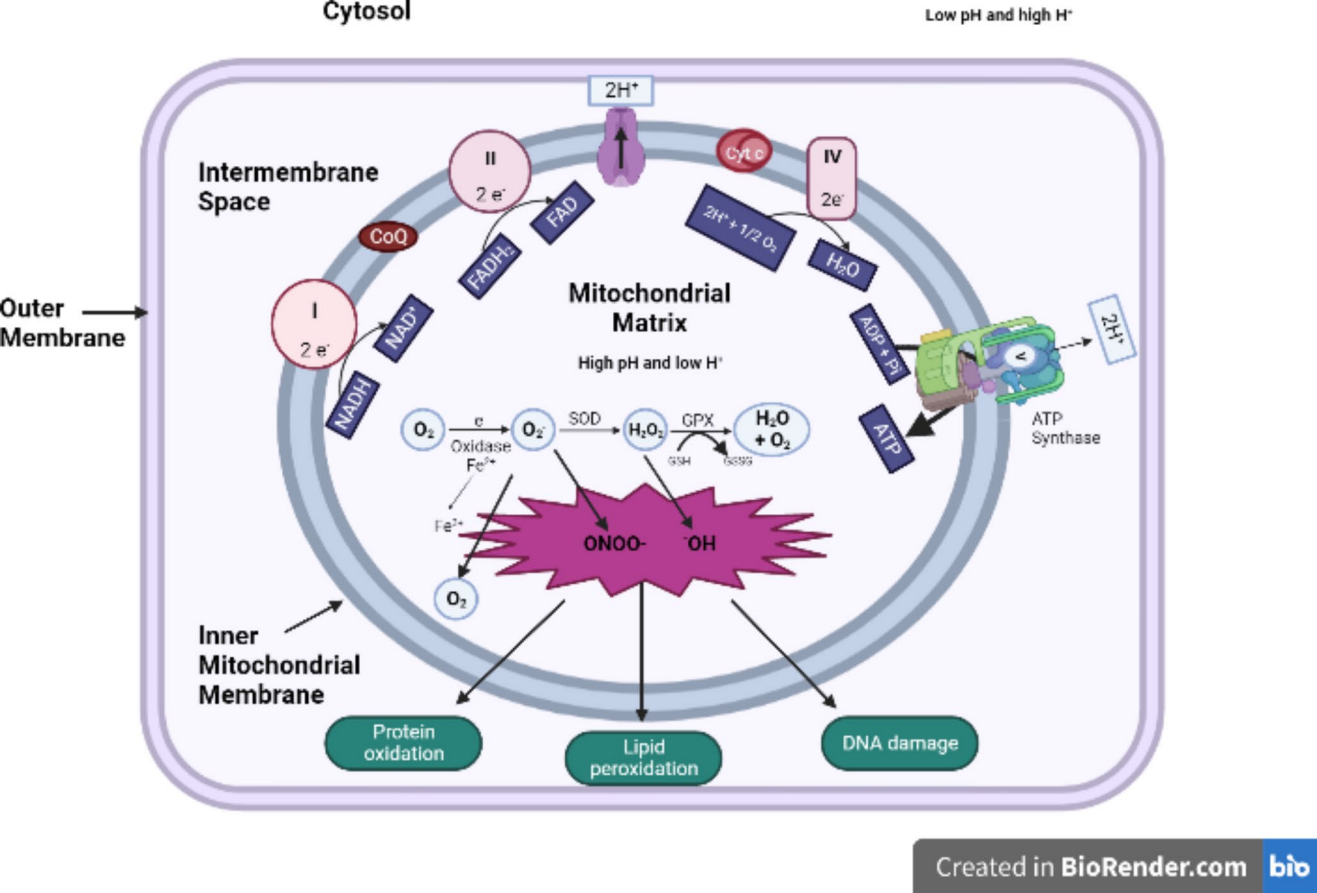
Electron transport chain (ETC) and generation of reactive oxygen species (ROS). Uncoupling of the normal electron transport chain marks the start of oxidative stress, where oxygen molecules, the main electron acceptors in the chain, gets converted into reactive oxidative species (ROS). The initial step involves the conversion of O_2_ to superoxide (^•^O_2_^−^). Superoxide dismutase (SOD) then turns it into hydrogen peroxide (H_2_O_2_), and the chain finally ends with the conversion of H_2_O_2_ to H_2_O by glutathione. Chiefly, superoxide and peroxide are the sources of more reactive species like ONOO- (reactive nitrogen species) and hydroxyl radicals (^−^OH), which are responsible for causing detrimental effects

At this juncture, a layperson would most likely ask, “What does mitochondrial dysfunction have to do with autism anyway?” but upon a closer inspection, the role of improper mitochondrial functioning in autism should be plenty clear. Multiple studies (Siddiqui et al. 2016)⁠ have revealed a potential correlation between the two (Fig. 3), with some studies suggesting that markers of mitochondrial function are deranged in ASD (Minshew et al. 1993), while others show that the activity of the electron transport chain (ETC) is affected, along with higher levels of oxidative damage being observed in the brains of autistic individuals. Interestingly, a meta-study has shown that around 5% of the children with autism exhibit mitochondrial disease, and biomarkers for the mitochondrial abnormality have been found in about 30–50% of them (Balachandar et al. 2021; Rossignol and Frye 2012).

ETC dysfunction, however, is not the only the only way in which problems with mitochondrial functioning can result in autistic phenotypes. Research has shown that calcium is also a key player in the same. For example, one study depicted how the transport rates through the mitochondrial aspartate/glutamate channel (AGC), which is activated by divalent calcium ions, was significantly enhanced in patients with autism (Palmieri et al. 2010).

This readily shows that calcium ions are important in the propagation of abnormal mitochondrial functioning, which can in turn be linked to the fact that both calcium, and glutamate (which has an important role in maintaining calcium levels) are implicated in excitotoxic conditions. Additionally, the activity of the enzyme nicotinamide adenine dinucleotide (NADH) oxidase, which is important in the ETC, was found to be reduced in the lymphocytic mitochondria of autistic children, which could potentially indicate a derangement in the normal oxidative phosphorylation processes occurring in this organelle (Giulivi et al. 2010).

Another finding of relevance reported that the activity of pyruvate dehydrogenase, an enzyme that is important for normal respiratory processes which in turn translate to correct functioning of the electron transport chain, was significantly decreased in the ASD group as compared to the control group (Giulivi et al. 2010; Weissman et al. 2008)⁠ This can have implications on ATP generation, and these results are one among multiple others which suggest a correlation between pyruvate levels and autism symptoms.

Moreover, the degree of mutations and deletions in the genes and DNA present in the mitochondria has also been noted to be higher in autistic children, further shedding light on the possibility of problems in the development of mitochondria. Incidentally, the functioning of certain complexes in the ETC, especially complexes III and V, was found to be decreased in the cerebellum of children aged 4–10 years of age on the spectrum (Chauhan et al. 2011), while that of all complexes, especially complex I, were lower in the frontal cortex.

Also, three of these complexes, including complex II, III, and V, were lower in the temporal cortex of these children. Similar results were, however, not obtained in the 14–39 years age group, indicating that developmental changes in the ETC in ASD were brain region-specific (Chauhan et al. 2011). At the same time, a different study found that complex IV levels were actually higher in ASD patients (Palmieri et al. 2010). Either way, these changes depict different forms of aberration in normal mitochondrial behavior, and the same has been tied rather strongly to the symptoms observed in autism spectrum disorders.

But of course, this mitochondrial dysfunction alone isn’t the reason behind the plethora of symptoms observed in autistic individuals, because oxidative stress is a much more serious implication arising from the same, as discussed above briefly. Increased levels of ROS and impaired respiratory chain activity have been noted to occur in the same regions of the brain. For example, the concentrations of markers of oxidative stress in are elevated ASD patients in the temporal cortex (Tang et al. 2013), a region where ETC abnormalities also frequently occur.

Moreover, the genes coding for some biochemical markers of mitochondrial dysfunction such as pyruvate, alanine, ubiquinone, carnitine, acyl-carnitine, and ammonia, among others show mutations (especially deletions) in autistic patients (Balachandar et al. 2021), and such abnormalities have also been noted in the gut of these patients, possibly providing a link to the gastrointestinal complications occurring in them (Rose et al. 2017).

As additional evidence regarding these observations, the levels of superoxide dismutase 2 (SOD2), one of the chief antioxidant enzymes, are lowered in children with autism (Tang et al. 2013), while those of hydrogen peroxide were seen to be raised (Giulivi et al. 2010). Interestingly, superoxide is typically formed during abnormal metabolism of oxygen, which might be expected when ATP synthesis does not proceed correctly.

Both these changes, along with many others, are characteristic of oxidative stress, and indicate an inability of the antioxidant system to cope up with the elevated levels of ROS that accompany mitochondrial impairment. These findings are further supported by others that suggest an increase in the levels of oxidized glutathione, coinciding with low reduced glutathione levels (James et al. 2009), which also serve to show that antioxidant activity might be reduced.

The reason behind the same is that excessive accumulation of these species can lead to the disruption of cell structures, proteins and lipids, and can eventually result in cell death. This, in context of the CNS and ASD, refers to a decrease in the number of functional neurons and a decline in synaptic plasticity, which translates to improper neural behavior.

Glutamate-induced excitotoxicity (Rojas 2014), which is brought about chiefly through the involvement of calcium ions, is also known to cause mitochondrial damage by inducing destruction of cellular components, and this results in oxidative stress more often than not. Since excitotoxicity has been implicated in ASD as well, this finding becomes even more interesting considering the role that the ERK/MAPK cascade has to play in the same. As such, this can provide a valuable link to further back the observations that ERK is important in the pathophysiology of autism.

## Targeting the ERK pathway in neuroinflammation and neurodegeneration

As the ERK pathway is believed to have a role in inflammatory pathways, and seeing its plausible involvement in neurological disorders, it is reasonable to consider its potential as a player in neuroinflammation. And since neuroinflammatiom has been linked to several CNS disorders, the implication of this pathway in the same might be considered important.

For example, neuroinflammation has been found to be associated with the development of the symptoms pertaining to Alzheimer’s Disease (Fu et al. 2019), and the ERK pathway has been correlated to the same by promotion of pro-inflammatory processes in the microglia (Chen et al. 2019). And drugs which are being evaluated for their efficacy in the therapy of this disorder might be involved, at least indirectly, in blocking the signaling through this cascade. Additionally, agents like trametinib (Henry et al. 2020), which is an inhibitor of MEK, an upstream regulator of ERK, have also been shown to have a positive effect in attenuation neuroinflammatory processes coupled with the cognitive impairment seen in traumatic brain injury. Interestingly, this effect is linked to the inhibition of microglia-induced inflammation, which in turn, involves the ERK pathway as well. Other agents which have shown potential in blocking these inflammatory processes include tectorigenin (Lim et al. 2018)⁠ and dexmetomidine (Qiu et al. 2020)⁠ which acts partially by downregulating the ERK pathway, among others.

Apart from this, the role of ERK signaling in neurodegeneration has also been an avenue of interest for drug development. As an example, one might consider the potential of phenolic agents, particularly flavonoids, in targeting this pathway and blocking its signaling, as a potential therapeutic avenue for the treatment of neurodegenerative disorders, owing to their antioxidant properties (Farzaei et al. 2018). Bioflavonoids, present in fruits and plants, have been found to exert a protective, and in many cases, therapeutic effect against neurodegeneration. To further highlight the therapeutic potential of ERK inhibitors in such disorders, a drug belonging to the class, known as PD-901, has shown promise in inhibiting Tau hyperphosphorylation which is associated with Alzheimer’s Disease (Medina et al. 2019)⁠ Moreover, plastoquinonyl-decyltriphenylphosphonium, known more commonly as SkQ1, which is a mitochondria-targeted antioxidant, has also shown efficacy in preventing this hyperphosphorylation, once again through ERK inhibition (Muraleva et al. 2021).

Also, seeing the involvement of this cascade in amyotrophic lateral sclerosis (ALS), the use of ERK blockers like selumetinib and cobemetinib, which have otherwise been implicated in neurological disorders, has also been suggested in this neurodegenerative disorder (Albert-Gascó et al. 2020).

## Niclosamide: much more than a humble antihelminthic

**Fig. 4:**
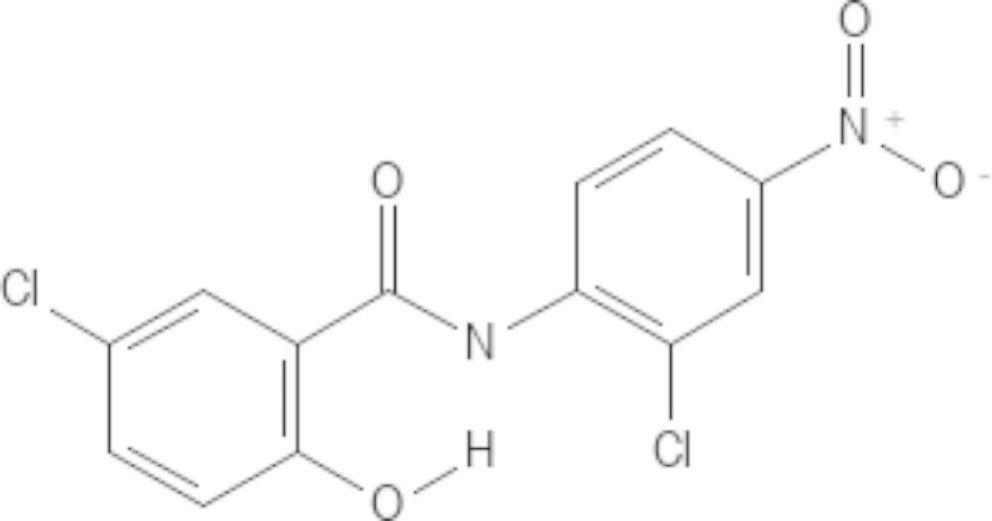
Structure of Niclosamide. (Drawn using PubChem Sketcher)

Niclosamide is an oral antihelminthic drug (Fig. 4) that has been approved by the FDA for the treatment of tapeworm infections and has been trusted for the same for over 50 years (Chen et al. 2018). The mechanism of action of this agent is the inhibition of mitochondrial oxidative phosphorylation (Frayha et al. 1997), as well as the production of ATP through anaerobic pathways. Beyond its use as an anti-infective, however, this molecule has found a range of applications in other diseases as well, from cancer to tuberculosis, as well as its potential as an immunomodulator.

First discovered in 1953, niclosamide has long been known to be effective in a host of different kinds of cancers (Kebebew et al. 2006; Wieland et al. 2013), as well as against the growth of mycobacteria, supporting its use as an anti-tubercular agent (Sun and Zhang 1999). Viral infections (Jurgeit et al. 2012) are also inhibited by this drug, and it has also found potential use in tackling metabolic syndrome (Kaur 2014), with a special emphasis on its efficacy on nonalcoholic fatty liver disease and diabetes mellitus type-2.

It typically acts in the capacity of an immunosuppressant and has the potential to reduce the expansion of helper T cells (Jang et al. 2021), resulting in alleviation of the stress induced by inflammatory processes, and the same has been evaluated through studies spanning diseases such as sclerodermatous graft-versus-host disease (Morin et al. 2016), rheumatoid arthritis (Huang et al. 2016), and systemic sclerosis (Cerles et al., 2016).

The pathways through which niclosamide exerts this myriad of effects are numerous and varied, but one specific signaling cascade that has been said to be involved in mediating the activity of this molecule is the MAPK/ERK cascade, and through the subsequent sections of this article, we hope to shed light on the possible ways in which this interaction between ERK and niclosamide is brought about, and how the same exerts an effect on neuronal plasticity, and by extension, can be related to ASD.

### Niclosamide as a therapeutic in neurological disorders

Apart from its conventional role as an anti-helminthic and its role in other diseases, particularly cancers, niclosamide has also been investigated as a potential neuroprotective agent. It has been found to reduce the incidence of apoptosis induced by ubiquitination of faulty proteins (Cheng et al. 2017). This is important because this apoptosis is known to play a role in different types of cancers, and is also implicated in neurodegenerative diseases like Parkinson’s Disease.

More importantly though, a recent study has found that this drug and its analogues can bring about the proper activation the PTEN-induced protein kinase 1 (PINK1) gene, a gene whose function is known to be hampered in implicated in autosomal recessive early-onset Parkinson’s Disease (Barini et al. 2018). Aberrations in this gene impair its catalytic activity, and it is believed that enhancing its activation can improve the symptoms of PD. Niclosamide can activate this enzyme by reversibly inhibiting mitochondrial membrane potential, and hence may have a future as a therapeutic agent in PD.

## Niclosamide and the ERK pathway

Niclosamide has been studied in multiple diseases and conditions apart from helminth infections, and among them, its potential as an anticancer agent has been very widely studied. And its usefulness in acting as an adjuvant therapeutic in cancers such as those of the ovary, colon, lung, and brain has been attributed to its capacity to regulate two major signaling pathways, namely the MAPK/ERK and the PI3K/Akt pathways. Since these cascades have also been established to be involved in the pathogenesis of autism spectrum disorders, there might be reason to believe that niclosamide can become a potential candidate for managing the progression and symptoms of this disorder.

One study (Cheng et al. 2017)⁠ which was carried out to assess the efficacy of niclosamide in glioblastoma has suggested that the drug can bring about apoptosis of cells, and also induces ubiquitination and subsequent degradation of proteins through stimulation of the ubiquitin-proteasome system (UPS). Since abnormal functioning of this system is implicated in disorders associated to ASD such as Angelmann syndrome, It might be suggested that niclosamide can also putatively result in partial alleviation of these disorders by restoring the normal functioning of protein ubiquitination.

More importantly, however, the same study also reported that the signaling cascade involving the MAPK/ERK pathway is inhibited by niclosamide, contrasting with previous research which has claimed that the drug shows little effect on the signaling. In this study, Western blotting techniques were used to determine the outcome of niclosamide use on ERK levels, specifically those of activated (phosphorylated) ERK and total ERK (active + inactive).

The results showed a time-dependent suppression of expression and phosphorylation in cancer cell lines, leading to the hypothesis that niclosamide has an inhibitory effect on the upstream regulators of the ERK cascade, while also preventing the adequate functioning of their downstream mediators.

Other evidence that has backed the effect that niclosamide can possibly have on the ERK cascade includes a study assessing the ability of the drug to inhibit the pro-inflammatory cytokine releasing-capacity of lipopolysaccharide (LPS)-induced mouse bone marrow dendritic cells (DC).

In this study (Wu et al. 2014), it was found that niclosamide can potentially block the LPS-induced activation of NF-KB as well as the c-Jun N-terminal kinase (JNK) and ERK pathways in dendritic cells, which might in turn be considered responsible for exerting a negative impact on DC activation. And since the ERK cascade has been implicated in the pathogenesis of ASD as well as DC induction, the fact that niclosamide can inhibit this pathway in the latter might provide valuable proof for its potential use in the latter.

Additionally, the anti-helminthic agent has also been found to have a negative regulatory effect on the activity of Ras (Ahn et al. 2017), and the same might further be linked to inhibition of the ERK pathway. As such, it might be suggested that niclosamide exerts its effect on the cascade at multiple steps in a much more complicated manner than earlier expected, instead of solely acting on one single mediator (ERK).

Finally, a study in osteosarcoma cell lines (Yeh et al. 2022)⁠ has revealed that niclosamide, when used in concentrations less than or equal to 200nM, can prevent the phosphorylation of ERK 1/2, and administration as a combination therapy with MEK inhibitors produced a significant reduction in the expression of tumor growth factor beta-induced protein (TGFBI), the marker that was chiefly employed in the determination.

**Fig. 5:**
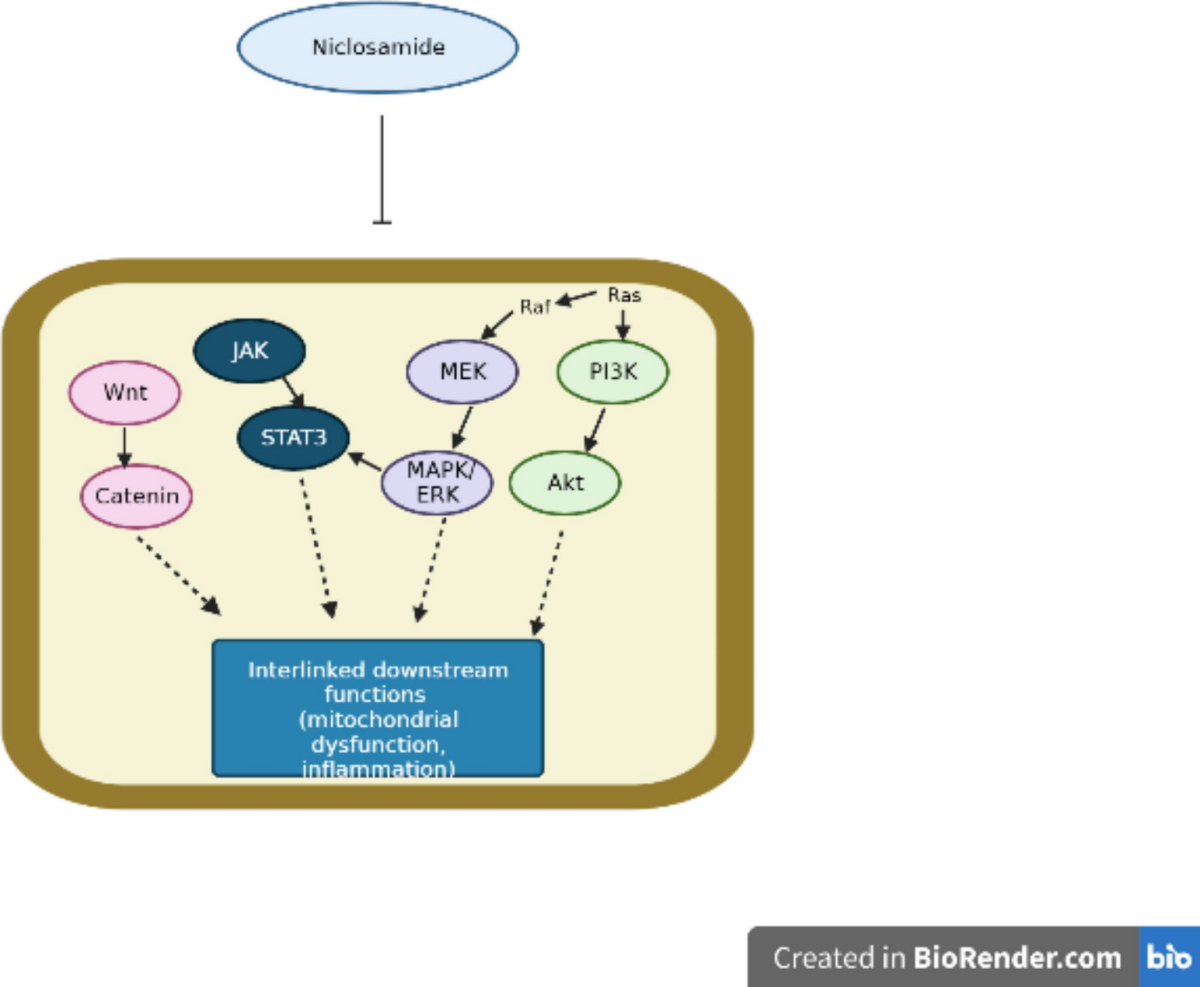
Effect of Niclosamide on ERK/MAPK and related pathways. Niclosamide has been shown to act on a number of interlinked signaling pathways, of which, the ERK/MAPK pathway is one. While it is believed that this drug does inhibit the ERK cascade, this effect might not always be direct and may be exerted indirectly by inhibition of other pathways

While the same has not yet been validated through animal studies, there is reason to believe that niclosamide might have a beneficial effect in alleviating the symptoms that are presented by patients on the autism spectrum, especially because of its potentially negative impact on the ERK signaling pathway and its phosphorylation/activation. Additionally, inhibition of other correlated pathways might also exert a similar effect on ERK, leading to its downregulation (Fig. 5).

For example, a transcriptomic meta-analyses (Forés-Martos et al. 2019)⁠ that was carried out in autistic brains has reported that the genes expressed, as well as the abnormalities observed in biological pathways, are similar to those seen in cancer. In detail, the meta-analysis involved the assessment of the gene expression profiles of tissues from the frontal cortex of ASD patients, and their comparison with the profiles of as many as 22 cancer types. Additionally, drug set-based overlaps were also studied between the two.

And the results obtained from the same showed that the expression profiles of the genes expressed in cancers of the brain, kidney, thyroid, and pancreas displayed a significant overlap of abnormalities with the expression profiles of ASD-related genes. At the same time, prostate and lung cancers were found to have expression profiles that were significantly deregulated in a direction that was opposite from ASD. What this means is that the genes that are expressed or whose expression is disrupted in case of these cancers are those that are known to have a potentially positive effect on ASD symptoms.

Among the pathways that were studied, a significant number were found to be impaired in both diseases, and primary among them are the impairments seen in the immune system, ATP synthesis, and oxidative phosphorylation. More importantly, it was also noted that kidney and brain cancers depict transcriptomic abnormalities in the regulation of the PI3K/Akt cascade, which is well-known to be associated to both, the ERK pathway and its role in autism, and the symptoms of ASD in general.

As such, the conclusion drawn from these meta-analyses led to the suggestion that the symptoms of autism spectrum disorder might have a direct comorbidity with cancers of the kidney, brain, pancreas and thyroid, while on the other hand were prostate and lung cancers which might be thought to have an inverse comorbidity with ASD. This type of related (or differential) expression of genes might in turn be the result of dysregulations in specific gene sets, all of which play roles in biological pathways and processes. Among them are perturbations in cascades known to be involved in immune function, cell cycle, energy metabolism, as well as the PI3K and G-protein linked signaling pathways.

Interestingly, the same study also revealed that niclosamide has the capacity to inhibit STAT signaling, and this has the effect of inducing differential expression of genes in certain cancers, which incidentally mimics the ASD-differentially expressed gene (DEG) signature. At the same time, the differential expression of genes in certain other cancers was reversed.

This inhibition of the STAT pathway might be important if one takes into account the fact that the Janus kinase (JAK)/STAT signaling cascade (Rawlings et al. 2004)⁠ can also be integrated with the MAPK/ERK cascade, and as such, the latter can eventually be subjected to inhibition by niclosamide occurring through the initial inhibition of the JAK/STAT pathway. How this happens involves a complex interaction of multiple mediators, and typically begins with growth factor receptor-bound protein (Grb2), which is a protein heavily implicated in ERK signaling.

This Grb2 molecule is known to contain a Src homology 2 (SH2) domain, which allows it to interact with receptors that have been phosphorylated by JAK. This interaction further brings about the activation of Grb2, and causes the downstream activation of the MAPK/ERK pathway. As such, JAKs may be at least indirectly involved in MAPK activation (Winston and Hunter 1996). One additional way in which the two cascades might be interlinked is through the phosphorylating activity that MAPK has on STAT, which in turn leads to a reduction in STAT activation (Jain et al. 1998). So, while niclosamide may or may not have a direct effect on the ERK pathway, it might be suggested to have at least a partial impact on ERK signaling by causing the blockade of STAT signaling.

Finally, while this is not directly correlated to the ERK system, niclosamide has also shown efficacy in inhibiting the p38/MAPK cascade, in a model of doxorubicin-induced muscle wasting. In this particular model, the drug prevented muscular atrophy commonly linked to doxorubicin, and believed to involve the functioning of p38. Interestingly, the ERK pathway is also involved in this side effect, and may hint at some effect of niclosamide on the same as well (Zhan et al. 2020). Additionally, inhibition of the ERK/Mnk1/eIF4E by this agent enhances dasatinib sensitivity in chronic myeloid leukemia (CML) (Liu et al. 2016). Niclosamide has also been found to have an inhibitory effect on the Wnt/ β-catenin pathway, akin to the effect it shows on the ERK, PI3K and STAT3 pathways (Cheng et al. 2017). And since the functioning of these multiple pathways often happens to be interlinked owing to their common upstream mediators, there is reason to believe that the inhibitory effect that niclosamide exerts on say, the PI3K, STAT3 or Wnt pathways might also lead to a related inhibition of ERK signaling.

### Niclosamide against mitochondrial dysfunction and oxidative stress

Importantly, apart from showing an effect on the ERK pathway itself, niclosamide has also been shown to have a significant effect on mitochondrial functioning, especially on the electron transport chain. While multiple studies have reported its efficacy on altering mitochondrial function in the therapy of different cancers, particularly in combination with other conventional anti-cancer agents, there have also emerged findings suggesting that this drug can prevent or ameliorate mitochondrial dysfunction caused by certain factors.

For example, one study has shown that niclosamide has the capacity to reduce the thermal hypersensitivity induced by paclitaxel in a model of peripheral neuropathy. Paclitaxel is widely known to induce nerve damage, and mitochondrial dysfunction mediated by the underexpression of the PINK1 gene is apparently an important player in the same (Jang et al. 2022). Since niclosamide can induce this gene expression discussed previously, there is reason to believe its beneficial effect in the model is attributable to prevention of mitochondrial abnormalities. This might be backed by another study involving a patented product with niclosamide as the active ingredient, which has shown efficacy in non small-cell lung cancer through a variety of mechanisms, with one of them being inhibition of mitochondrial dysfunction (Kim et al. 2017).

Moreover, a comparative animal study of niclosamide against vitamin C on methotrexate-induced hepatotoxicity has also reported the potential of this antihelminthic on preventing oxidative stress and bringing down the levels of ROS in cells (Zeki and Al-Gareeb 2021). These findings, if taken together with the effect that niclosamide shows in inhibiting the ERK/MAPK pathway and its attending inflammation, can suggest the possibility of this drug having a potential efficacy in tackling ERK-induced mitochondrial dysfunction and the associated oxidative stress, which can support its usefulness in CNS disorders, and provide a basis for repurposing it towards autism spectrum disorders.

## Niclosamide as a potential therapeutic agent for autism spectrum disorders: a strong hypothesis

From the above discussion, one might conclude that niclosamide, owing to its potential in the prevention or as a therapeutic agent in mitochondrial dysfunction, and by extension, in conditions pertaining to oxidative stress, might also show efficacy in the treatment of autism spectrum disorder. This can be said based on the effect that mitochondrial abnormalities, occurring chiefly during neuroinflammatory processes, has on neuronal health and development, especially in neonatal brains. Additionally, these abnormalities are, more often than not, linked to the generation of reactive oxygen species, which can in turn bring about more severe neuronal damage, giving rise to the neuronal aberrations and neurodevelopmental/neurodegenerative sequelae which are representative of ASD, among other diseases. And since the neuroinflammatory activity of ERK has been implicated to have a bearing on these secondary processes, the ERK-inhibiting activity of niclosamide might make it a plausible therapeutic agent for the treatment of ASD and its related symptoms. And this effect might primarily be achieved through the blockade of neuroinflammation, and a reduction in oxidative stress arising due to mitochondrial dysfunction. As such, further research into the potential of this antihelminthic in the therapy of autism spectrum disorders might bring out promising results.

### Future prospects

With the incidence of autism spectrum disorders on the rise in multiple parts of the world, the need for newer therapies that focus on the underlying cause of the disorder instead of solely on symptomatic relief has also increased. And since mitochondrial dysfunction and oxidative stress have been found to be key players in the pathogenesis of ASD, drugs that alleviate these abnormalities might prove to be useful in the long run. While several pathways are known to be involved in the regulation of mitochondrial function, the role of the ERK/MAPK pathway is of interest. As such, niclosamide, which is known to act on and inhibit the signaling through this pathway, and has been utilized previously in other neurological disorders, might be a potential candidate for repurposing for the treatment of ASD. This is especially true seeing as how this agent has already been concluded to have a bearing on mitochondrial function, and has produced encouraging results against neuronal damage. So, niclosamide might have a future as a therapeutic agent in autism. However, a strong pre-clinical and clinical proof of concept needs to be established.

## Conclusion

Autism spectrum disorder (ASD) is a neurodevelopmental disorder involving the complex interplay of genetic and environmental factors. Nevertheless, the etiology of this disorder remains largely unknown, but a number of potential factors and agents have been implicated as having a bearing on the pathogenesis of autistic symptoms. Over the years, improper functioning of certain physiological pathways have been shown to be involved in giving rise to these symptoms, and multiple toxicants which act by tipping the balance of these pathways have also been identified. Among them, one pathway that has been taking center stage is the ERK pathway, owing to its myriad of effects, both beneficial and detrimental, on the health of the central nervous system, and on the pathophysiology of autism. While low functioning of the ERK cascade is responsible for problems with synapse generation, elevated functioning can have toxic effects as well, especially through routes which involve mitochondrial damage and oxidative stress. As such, drugs which act by inhibiting the overactivity of this pathway can afford benefit, though at present, the same has not been studied in depth. Niclosamide, an antihelminthic and immunomodulator agent has been shown to have an inhibitory effect on elevated ERK functioning, with the same having been validated in cancer models. But given the suspected role of ERK in the development of autism, this molecule might have a potential therapeutic use in combating the symptoms of the disorder.

## Data Availability

All the data has been included.
